# Geographical distribution of human pythiosis in the USA

**DOI:** 10.1128/spectrum.02103-24

**Published:** 2024-12-06

**Authors:** Raquel Vilela, Sue C. Grady, Priscilla Rocha Vilela, Leonel Mendoza

**Affiliations:** 1 Federal University of Minas Gerais, Faculty of Pharmacy, Belo Horizonte, Minas Gerais, Brazil; 2 Biomedical Laboratory Diagnostics, East Lansing, Michigan, USA; 3 Department of Geography, Environment, and Spatial Sciences, Michigan State University, East Lansing, Michigan, USA; 4 Department of Microbiology, Genetics and Immunology, Michigan State University, East Lansing, Michigan, USA; Institut Pasteur, Paris, France

**Keywords:** human pythiosis, *Pythium insidiosum*, *P. periculosum*, *P. aphanidermatum*, density geographic distribution

## Abstract

For more than 100 years, the infections caused by *Pythium insidiosum* have been well documented in horses. However, recently, pythiosis was also described in several species, including dogs, cats, cattle, zoo captive animals, and humans. Human pythiosis is a life-threatening infection requiring an early diagnosis for a successful management. The clinical picture of pythiosis mirrors the clinical features of infections caused by other pathogens, including filamentous fungi, making its diagnosis challenging. This fact had led to several misdiagnosed cases involving filamentous fungal pathogens. The first pythiosis human case in the USA was reported in 1989. Soon after, various pythiosis cases of orbital disease in children and on the limbs of adults were sporadically diagnosed. To investigate the geographical distribution of human pythiosis in the USA, we constructed a density USA map using a recent report of pythiosis in animals highlighting the known human cases. Our data showed that *P. insidiosum* human infections in the USA are diagnosed in the same geographic areas where animal pythiosis occur. Physicians handling clinical cases like those so far reported can use the geographic distribution of pythiosis human cases in this study as a guide in their clinical diagnosis and in the early management of putative cases.

**IMPORTANCE:**

The relevance of the study not only resides in the epidemiological distribution of animal human pythiosis in the USA (our team’s recent publication), but also shows physicians the areas where *Pythium insidiosum* thrive in endemic areas. Our manuscript shows to the medical and veterinary communities the current areas of human and animal pythiosis, an event that could have a direct impact on the epidemiology, diagnosis, and management of pythiosis.

## INTRODUCTION

Pythiosis is a disease of humans and animals caused by at least three different *Pythium* species, *P. aphanidermatum*, *P. insidiosum*, and *P. periculosum* (1–4). However, the most prevalent species infecting mammalian hosts in the USA are *P. insidiosum* (5). These oomycetous fungal-like pathogens belong to the Stramenopiles, Alveolates, and Rhizaria supergroup located in a different phylogenetic cluster from the fungi (6). These mammalian pathogenic species are found in wet environments of tropical and subtropical countries (endemic areas). Susceptible human and animal species get in contact with their infecting propagules (zoospores, hyphal elements) through small skin cuts (5, 7). Pathogenic *Pythium* species rapidly evolved developing skin ulcerated tissues and, in some cases, dissemination to blood vessels (7). If the disease is not rapidly diagnosed, it could become life-threatening with high morbidity and mortality rates (5, 7). Human pythiosis is more prevalent in Asia, with few cases diagnosed in other world areas, including the USA (7).

The first USA human case of pythiosis with proven culture was reported in 1989 by Rinaldi et al. (8) in a 4 1/2 year-old boy with orbital infection and no previous underlying disease. The boy was accidentally stroked in the right eye by the elbow of his sister, developing orbital swelling that progressed in the following days. *Pythium insidiosum* was isolated in pure culture, and the boy was successfully treated by radical surgery, losing his right eye in the process. A retrospective review of the USA literature detailed several misdiagnosed cases of orbital fungal infection in children as putative cases of pythiosis was also investigated (9). The study based its finding on the histopathological description of the cases showing strong eosinophilic response around sparsely septate hyphae. This study suggested that cases of orbital disease in USA children had been incorrectly attributed to phenotypically similar filamentous fungal pathogens (9). After Rinaldi et al. (8), reports of several cases of human pythiosis had been recorded; some of them appeared in various journals, whereas others remained unpublished (Table 1) (10–29). In the USA, orbital pythiosis in children is the main clinical feature of the disease, whereas in adults, the subcutaneous forms on limbs and other anatomical areas are common (Table 1). Intriguingly, cases diagnosed in India and Thailand affecting the eyes (keratitis) and/or the subcutaneous tissues, with some presenting dissemination to the blood vessels, are particularly common (7). Oddly, subcutaneous pythiosis with dissemination to the blood vessels is rarely diagnosed outside Asia (5, 7).

**TABLE 1:** Cases of human pythiosis diagnosed in the USA since 1967

Year	Host/disease	Reference	Isolate
1967^*c*^	Texas boy with aneurysm Zimmerman L.E. mentioned by Margo et al. (10)	Eosinophilia + hyphae. Read before the Vehoeff Society Meeting, 24–26 April 1967.	None
1969^*c*^	Iowa boy, orbital disease after trip to Texas	Blodi et al. (11)	None
1969^*c*^	Texas boy, aneurysm secondary to orbital disease	Blodi et al. (11)	None
1976^*c*^	Florida boy, orbital disease	Morris and Spock (12)	None
1978^*c*^	Florida boy, orbital disease	Margo et al. (10)	Lost
1981^*c*^	Arizona boy orbital disease	Whithurst and Liston (13)	None
1988	Texas boy, orbital disease, first case with culture	Rinaldi et al. (8)	Lost
1989	Woman, corneal ulcer, infected in Haiti	Virgil et al. (14)	ATCC 76049
1989^*d*^	Texas man, spider bite leg	Unpublished. Dr. A.A. Padhye, Southwest Texas Methodist Hosp.	ATCC 90586
1997	Tennessee boy, orbital disease	Shenep et al. (15)	ATCC 20269; MTPI-10^*a*^
1998^*d*^	Philadelphia man, keratitis	Unpublished (mentioned by Schurko et al., 2003) (16, 17)	Lost
2001	New York boy, pleuro-pericarditis	Heath et al. (18)	Lost
2003	Virginia woman, subcutaneous	Unpublished, Dr. Tim Straight, Walter Reed Army M.C.	Lost
2008^*b*^	Illinois man, systemic infection	Unpublished. Dr. Elizabeth Henry, Loyola Uni Med Center	MTPI-33^*a*^
2008^*b*^^,^^*d*^	Mexican girl with extensive burns, diagnosed in Texas	Franco et al. (19)	MTPI-36^*a*^
2010^*b*^^,^^*d*^	USA man infected in Israel, diagnosed in Massachusetts	Dr. Thanhelhco et al. (20)	MTPI-35^*a*^ *Pythium periculosum*
2011^*b*^	Texas girl, leg infection	Salipante et al. (21)	MTPI-43^*a*^
2011	Nebraska boy, leg infection	Hoffman et al. (22)	Lost
2012^*b*^	Tennessee girl, orbital disease	Kirzhner et al. (23)	MTPI-28^*a*^
2013^*b*^^,^^*d*^	Caribbean man, arterial, diagnosed in Virginia	Pan et al. (24)	MTPI-48^*a*^
2013	Wisconsin girl, disseminated infection	Schloemer et al. (25)	Lost
2016	Florida man, subcutaneous right leg	Hilton et al. (26)	None
2017^*b*^	Illinois man, subcutaneous right leg	Roy et al. (27)	None
2018^*b*^	Texas girl, orbital disease	Unpublished, Dr. Amanda Evans, Pediatrics UT Southwestern Med Center	MTPI-62^*a*^
2018	Texas man, infected in hot spring in New Mexico	Perkins et al. (28)	Lost
2018^*d*^	USA man, keratitis acquired during a trip to Costa Rica	Neufeld et al. (29)	None
2019^*b*^	Georgia girl, abdominal subcutaneous	Unpublished, Dr. Stephen Thacker, Children Hospital of Savanna, GA	MTPI-69^*a*^
2021^*b*^	Florida man, subcutaneous leg infection	Unpublished, Dr. Ahn and Dr. Missall U. of Florida Health, FL	None
2023^*b*^	Texan boy, arm infection	Unpublished, Dr. Dan Chang, Southwestern Med. Center, Dallas	MTPI-102^*a*^
2023^*b*^	Oklahoma girl, orbital disease	Unpublished, Dr. Thai Do, Oklahoma Eye Hospital	MTPI-103^*a*^

^*a*^ MTPI: Biomedical Laboratory Diagnostics, Michigan State University (MSU) *Pythium insidiosum* collection.

^*b*^ Human cases from the MSU diagnostic laboratory were involved.

^*c*^ Putative cases selected as cases of pythiosis based on their clinical, histopathological, and, in some cases, their cultural traits (8).

^*d*^ Imported cases diagnosed in the USA.

Details on the global aspects of human and animal pythiosis were recently reviewed (7). However, only human USA cases so far found in the literature were mentioned. Therefore, the objective of this study is to disclose the geographic location of published and unpublished pythiosis human cases diagnosed in the USA (Table 1). To achieve this goal, the known human cases were placed on a density USA map constructed with the animal data (cats, dogs, horses, and other animal species) collected by Nguyen et al. (30).

## MATERIALS AND METHODS

### Pythiosis cases in the USA

The ZIP codes (places where the infection was originally diagnosed) collected by Nguyen et al. (30) were used to construct a USA density map displaying the heavy areas in which cases of pythiosis in animals occurred (see below). To our knowledge, at least 30 cases of human pythiosis in the USA had so far been reported, including six individuals that acquired their infections overseas, but diagnosed in the USA (Table 1) (7). The pythiosis human cases’ ZIP codes from Nguyen et al. (30) were collected and displayed with color dots on a USA density map constructed with the available pythiosis animal data (30).

### Human and animal pythiosis case density map

According to Nguyen et al. (30), between 2000 and 2016, there were 1,676 confirmed cases of animal pythiosis diagnosed on their farms or not far away from their farms. To identify the geographical areas where the animal cases occurred, the ZIP codes, where animal pythiosis was originally diagnosed, were used as a geographic reference. Mapping and spatial analysis in ArcGIS v.8.5 (Environmental Systems Research, Inc.) (31, 32) were carried out using Nguyen et al.’s (30) published data. The 2014 ZCTA (ZIP code tabulation areas) boundaries were the base ZIP code layer by which ZIP codes that changed over time were recoded (31). There were 101 cases with ZIP codes that were recoded to the 2014 base file. There were also eight cases with ZIP codes that could not be matched and, thus, were removed from the spatial data set. The final data set contained 1,666 ZIP codes corresponding to previously reported cases of animal with pythiosis in 36 states (30).

The density of cases involving *P. insidiosum* infection per square kilometer (km^2^) was calculated by summing the number of cases to the centroid of each ZIP code and using these point data to implement a kernel density function in ArcGIS v. 8.5 (31). The state boundaries and a graticule of lines of latitude and longitude were overlaid for spatial reference (32). The ZIP codes (original place where the cases were first diagnosed) of 30 proven pythiosis human cases (Table 1) were overlaid at the ZIP code centroid onto the density map of animal pythiosis to analyze their spatial relationships within the map as per Manson et al., (32).

## RESULTS

**Fig 1:**
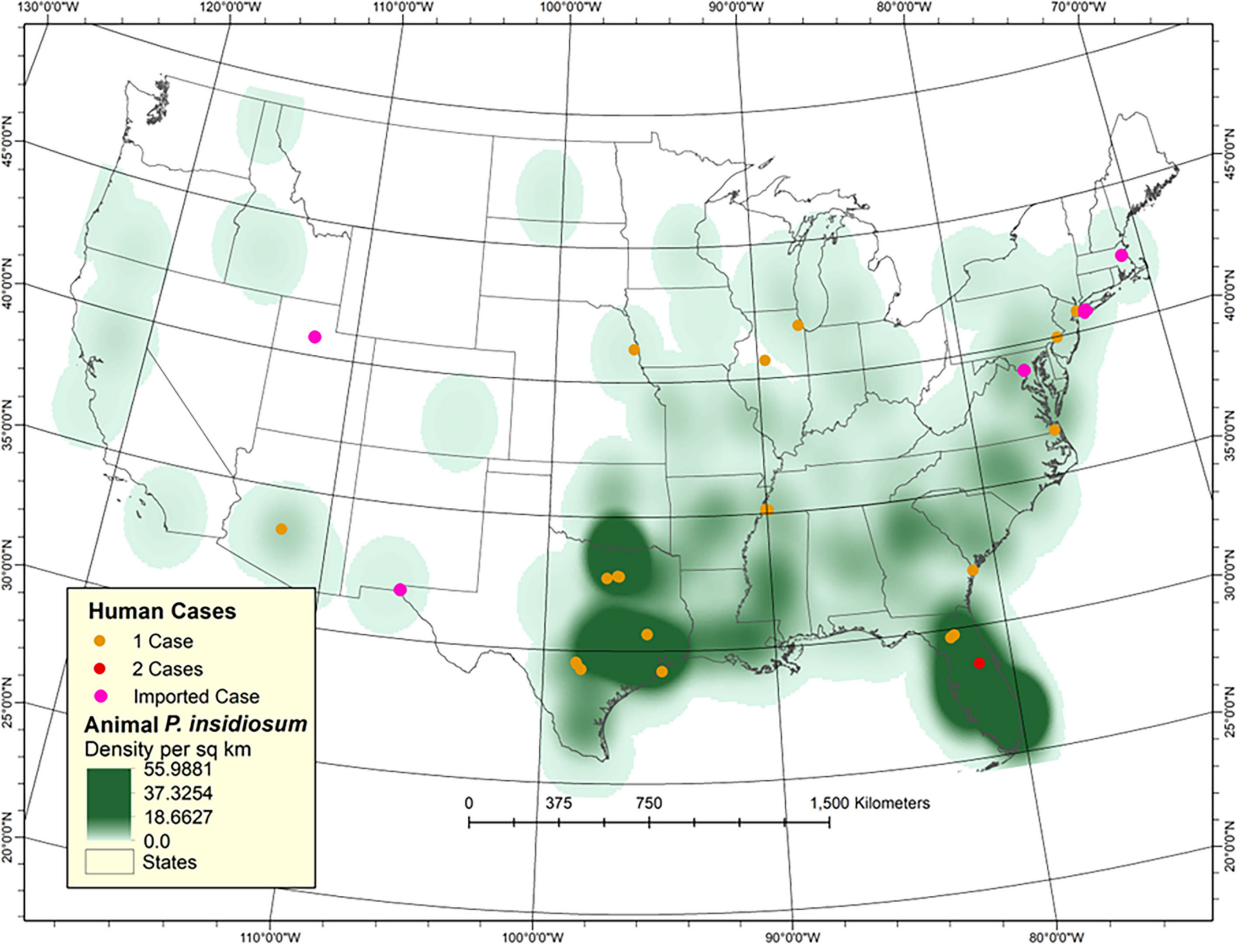
Spatial distribution of animal (green areas) and human (orange and red dots = autochthonous USA cases, purple dots = imported cases) pythiosis cases diagnosed in the USA from 1967 to 2023 and displayed on a density map (map source: Department of Geography, Michigan State University).

Animal pythiosis cases on the density map were found in high numbers in USA’s central and east coasts, particularly in the states bordering the Gulf of Mexico (Fig. 1). The map shows Florida and Texas with the highest number of cases of animal pythiosis, followed by Alabama, Arkansas, Georgia, Louisiana, Mississippi, and Oklahoma (Fig. 1). The map showed human pythiosis cases that occurred in eastern states, such as South Carolina, Tennessee, as well as northern states, including Illinois, Indiana, Kentucky, New York, North Carolina, Ohio, Pennsylvania, Virginia, Wisconsin, and nearby states (Fig. 1). In Texas, most animal cases occurred in the eastern section of the state, with no cases recorded in the western section and two sections split up at longitude 100°0′0″W (Fig. 1). Few animal cases were found in the eastern section of the map, with most of the cases located in Arizona, New Mexico, and Southern California.

The ZIP codes of the 30 diagnosed USA human cases were found in the states where the higher number of pythiosis animal cases occurred (Fig. 1, orange, pink, and red dots). This includes Florida (five cases), Illinois (two cases), Tennessee (two cases), and Texas with the higher number of pythiosis human cases (eight cases), with the remaining human cases distributed in the geographical areas where previous cases of animal pythiosis are usually found (Fig. 1; Table 1). It is important to note that some human cases occurred in states with few animal cases (Arizona, Nebraska) and states along the east Atlantic coast (New York, North Carolina, Pennsylvania) (Fig. 1). No human cases have been reported so far on the Pacific West coast. In addition, six imported pythiosis human cases diagnosed in the USA were also included (Fig. 1, pink dots; Table 1).

## DISCUSSION

In the USA, human infections caused by *Pythium* spp. are rather sporadic, with a handful number of cases so far reported (Fig. 1; Table 1). In contrast, human pythiosis in India and Thailand is diagnosed with more frequency than in any other geographical area of the world, with the data contrasting with the low number of cases in lower animals in the same area (7, 33, 34). Oddly, in the USA, we see more cases in domestic animals than in humans. (5, 30, 33) Some investigators had postulated that a high percentage of individuals suffering from Thalassemia in Thailand, a disease that induces a high load of iron in blood, could be a predisposition factor for acquiring the disease (7, 34, 35). They also argued that in other countries, including the USA, cases of thalassemia are not found in high numbers and therefore, few human cases of pythiosis are expected in such areas (1, 7, 33–36). We believe that the true predisposition factors to *P. insidiosum* infection in susceptible individuals are not well understood. Although several hypotheses have been recently proposed (7, 35), all efforts have been affected by the fact that there is not a suitable animal model for investigating *P. insidiosum* key virulence factors (5, 33, 37).

Based on the report of pythiosis cases in the USA literature in several species, southern states, particularly Alabama, Florida, Louisiana, Mississippi, and Texas, are the heavy endemic areas of *P. insidiosum*, with sporadic cases in nearby states (5, 7, 30, 33). However, our study showed that the distribution of the disease is not the same in all states. An example is Texas. Texas is second to Florida in the number of cases of different species. However, the generated map showed the diagnosed cases in this state occurred only in the eastern section of the state (Fig. 1). So far, no cases west to latitude 100°W, a very dry section of the state, have been recorded. Since *P. insidiosum* requires water to complete its life cycle in nature (31, 36), this is probably the main reason why in Texas, we do not see cases west to 100°W latitude, and the cases we see in the eastern section of the state occurred frequently in wet summer months (5, 30, 33). This observation is also true for all northern states west to the 100°W latitude. With the exception of North Dakota with few cases of pythiosis in animals, a distinctive line with few cases can be observed west to latitude 100°W (Fig. 1). Cases reported in Arizona and New Mexico (arid areas) in dogs, horses and humans correspond to areas with plenty of water or nearby golf courses. A contrasting example is Florida. In this state, cases of the infection by *P. insidiosum* are well distributed throughout the state (Fig. 1). This was an expected outcome since warms wet environments have the ideal ecological elements for this oomycete to thrive in nature. The recent environmental recovery of *P. insidiosum* and *P. periculosum* from several lakes in central Florida and around the Atlantic coast presents examples of the wide distribution of these pathogenic oomycetes in several states, supporting the results of this study (38, 39).

Pythiosis cases in Northern States affecting several species (Illinois, Indiana, Kentucky, Minnesota, New York, Ohio, Pennsylvania, Virginia, and Wisconsin) corroborated recent reports of the disease in humans as they appeared in several journals (7, 8, 14, 15, 18, 19, 21–26, 29). Of interest to this study was the case of a boy in Nebraska with leg infection in an area where few cases of animal pythiosis are usually diagnosed (Fig. 1). This case suggests that *P. insidiosum* is also present in the wet areas of northern USA. This finding and those in the literature (1, 7, 30, 33, 35, 36) imply that *P. insidiosum* colonize northern USA environments during summer months and likely survive the winter freezing temperatures that northern states are subjected to during the months of December to April.

Five imported cases caused by *P. insidiosum* and *P. periculosum* acquired overseas and diagnosed in the USA were included to illustrate that individuals visiting endemic areas (overseas or USA endemic areas) could return to states, these include few and no reported cases (Fig. 1 purple dots, Table 1). For instance, Blodi et al. (11) reported a case of pythiosis (originally diagnosed as a fungal infection) in a Utah boy after a trip to Texas. The overseas imported cases in this study include a corneal ulcer case from which *P. insidosum* was isolated in a Haiti woman (Table 1) (14). Another case was mentioned by Shurcko et al. (16, 17) in a man from Afghanistan, but residing in the USA. Because the isolate phylogenetic traits pointed to the Asia region, the authors speculated he probably acquired his infection after contact with paraphernalia from Afghanistan. Thanhelhco et al. (20) described a case in a USA man that acquired his infection after working in Israel’s wet areas and later diagnosed in Boston, USA. Incidentally, using molecular methodologies, the isolate recovered from this case was later identified as *P. periculosum*. This finding suggests that *P. periculosum* may be common in countries surrounding the Mediterranean Sea, a hypothesis supported by a recent a human Spain case and two Italian dogs from which the same species was recovered in culture (40, 41). Epidemiologically speaking, the Boston case is unique because human pythiosis cases from that region were unknown. In addition, our research laboratory was involved on an arterial pythiosis case in a Caribbean man (24). We were able to culture the pathogen (MTPI-48), but the man did not respond to antifungal treatment and later died (Table 1). Neufeld et al. (29) informed of the last known USA imported case. They described a severe case of keratitis in a USA man traveling on a vacation to Costa Rica (endemic area of horse pythiosis) and exposed to rivers and wet environments. After his return to Utah, he was diagnosed having an infection caused by *P. insidosum* (Fig. 1, Utah pink dot; Table 1). These cases illustrate that the epidemiology of pythiosis plays a key role in its putative diagnosis and, therefore, in the selection of the appropriate management (5, 7, 33–36).

This is the first study in the USA identifying the geographical areas using a density map, where the infections caused by the mammalian *Pythium* pathogenic species, including humans, occur. The objective of this epidemiological study was not only to show the areas where the human pythiosis are usually diagnosed, but also to call the attention to the medical and veterinary communities on the geographic location of *P. insidiosum* and *P. periculosum*. As physicians become familiar with the epidemiology of the infections caused by these fungal-like pathogens of humans and other animals, a rapid diagnosis could lead to a rapid management using antifungal, immunotherapy, and other therapeutic approaches (5, 7, 33, 34). We hope this study helps physicians to consider the possibility of infections caused by these pathogens in the highlighted geographical areas.

## Data Availability

The data from cases of animal pythiosis used to generate the density map are available at https://doi.org/10.2460/javma.20.10.0595.
